# The sensibility of the new blood lipid indicator——atherogenic index of plasma (AIP) in menopausal women with coronary artery disease

**DOI:** 10.1186/s12944-020-01208-8

**Published:** 2020-02-24

**Authors:** Qianyun Guo, Shu Zhou, Xunxun Feng, Jiaqi Yang, Jiaming Qiao, Yingxin Zhao, Dongmei Shi, Yujie Zhou

**Affiliations:** Department of Cardiology, Beijing Anzhen Hospital, Capital Medical University, Beijing Institute of Heart Lung and Blood Vessel Disease, Beijing Key Laboratory of Precision Medicine of Coronary Atherosclerotic Disease, Clinical center for coronary heart disease, Capital Medical University, Beijing, 100029 China

**Keywords:** Atherogenic index of plasma, Coronary artery disease, Lipid, Postmenopausal women

## Abstract

**Background:**

Dyslipidemia is a key driver of coronary artery disease (CAD) development. This study aimed to determine whether the atherogenic index of plasma (AIP), a novel comprehensive lipid index, is an independent and reliable predictor of CAD risk in postmenopausal women.

**Methods:**

A cohort of consecutive 4644 postmenopausal women (aged 50 or above) undergoing coronary angiography (CAG) in Anzhen Hospital (Beijing, China) from January–December 2014 was included in the analysis. Of them, 3039 women were CAD patients, and 1605 were non-CAD subjects.

**Results:**

Relative to control subjects, TG levels in CAD patients were higher and HDL-C levels were lower. In CAD patients, non-traditional lipid profile values (TC/HDL-C, AI, and AIP) were significantly elevated relative to controls. AIP was positively correlated with TC (r = 0.157), TG (r = 0.835), LDL-C (r = 0.058), non-HDL-C (r = 0.337), TC/HDL-C (r = 0.683), LDL-C/HDL-C (r = 0.437), LCI (r = 0.662), and AI (r = 0.684), and negatively correlated with HDL-C (r = − 0.682) (all *P* < 0.001), but was independent of age (r = − 0.022; *P* = 0.130) and BMI (r = 0.020, *P* = 0.168). Aunivariate logistic regression analysis revealed AIP to be the measured lipid parameter most closely related to CAD, and its unadjusted odds ratio was 1.824 (95% CI: 1.467–2.267, *P* < 0.001). After adjusting for several CAD risk factors (age, BMI, smoking, drinking, EH, DM, hyperlipidemia, and family history of CVD, AIP was still found to represent a significant CAD risk factor (OR 1.553, 95% CI: 1.234–1.955, *P* < 0. 001).

**Conclusion:**

AIP may be a powerful independent predictor of CAD risk in Chinese Han postmenopausal women, and may be superior to the traditional lipid indices.

## Background

Coronary artery disease (CAD) is characterized by high prevalence and incidence, and is associated with one of the highest mortality rates worldwide [1]. With the economic development and lifestyle changes, such as increasing consumption of meat and decreasing the amount of physical exercise, CAD rates in China have continued to increase in recent years, and this condition has become the most severe disease affecting the mortality rate throughout the country [2]. From 2002 to 2016, the CAD-related mortality of urban residents increased from 39.6/100,000 to 113.5/100,000, and in rural residents from 27.6/100,000 to 118.4/100,000 in China [3]. Additionally, CAD-associated morbidities seriously negatively affect the health of affected individuals.

Dyslipidemia is among the best-understood CAD risk factors. Traditional lipid measurement indices (including Total Cholesterol (TC), Low-Density Lipoprotein Cholesterol(LDL-C), and Triglyceride (TG)) have been proven to be related to CAD onset. Many studies have demonstrated that the reduction in LDL-C significantly reduces cardiovascular event incidence rates. However, even after the recommended level of LDL-C is achieved, the residual cardiovascular risk remains at approximately 50%, highlighting the need to identify new predictors of CAD [4].

Composite lipid indices, including non-HDL-C (TC minus HDL-C), TC/HDL-C, LDL-C/HDL-C, non-HLD-C/HDL-C (atherosclerotic index, AI) and TC*TG*LDL/HDL-C (lipid comprehensive index, LCI) are thought to be better means of predicting CAD risk than single lipid parameters [5]. Recently, the atherosclerotic index of plasma (AIP), i.e., the logarithm of the value of plasma TG divided by value of plasma HDL, has gained recognition among the researchers. AIP may be an important predictor of atherosclerosis and cardiovascular disease, superior to the standard atherosclerotic lipid profile [6]. Epidemiological studies have demonstrated that there is a significant correlation between AIP and several CAD-related risk factors including obesity, essential hypertension (EH), and diabetes mellitus (DM) [7, 8]. In one study of patients in a hospital setting undergoing coronary angiography (CAG), of all measured lipid parameters AIP had the strongest association with CAD [9].

Menopause can have a significant impact on the social, reproductive, physical, and mental health of affected women. The level of lipids beneficial for the cardiovascular system in postmenopausal women is lower than in premenopausal women [10]. Several cross-sectional and longitudinal studies have suggested menopause to be associated with changes in CAD risk factors. Relative to premenopausal women, postmenopausal women have higher TC, LDL-C, VLDL-C, and TG plasma levels [11]. In a study performed in Cameroon, Nansseu and coworkers documented that AIP was unable to independently predict CAD risk in postmenopausal women [12]. In contrast, an investigation conducted in Xinjiang, China, has demonstrated the validity of AIP as a novel and independent predictor of CAD in postmenopausal women [13]. In another study of the Han Chinese, AIP was independently associated with acute coronary syndrome risk in men only [14]. Therefore, whether AIP can be used as a biomarker to predict CAD in postmenopausal women remains controversial. Moreover, it is unclear whether the value of AIP is associated with CAD risk in postmenopausal women in the Han Chinese population. Therefore, this study sought to explore the association AIP and CAD risk, and to explore whether AIP may be superior to other lipid indices as a means of predicting such CAD risk in postmenopausal women.

## Methods

### Study population

The present investigation was designed as a single-center observational study, which included postmenopausal women aged 50 or over who underwent CAG at the Anzhen Hospital (Beijing, China) between January 2014 and December 2014.

**Fig. 1:**
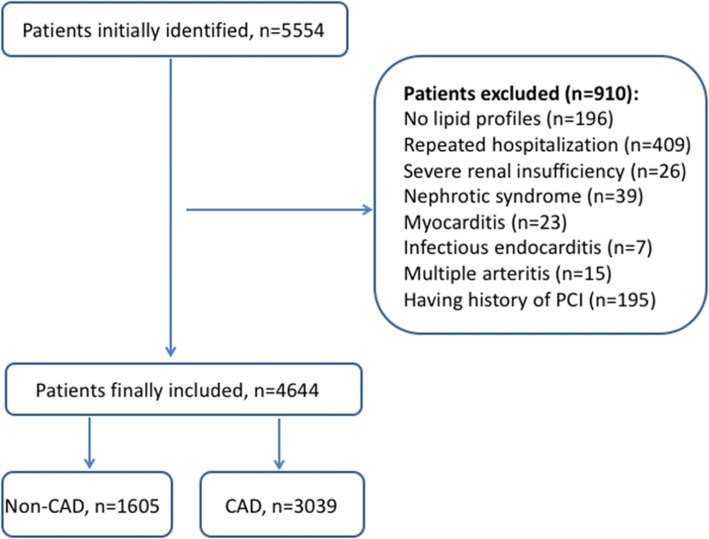
Flow chart illustrating the process of participant enrolled in the study

Patients were excluded from this study if they had been repeatedly hospitalized, if they suffered from myocarditis, infective endocarditis, polyarteritis, renal insufficiency, nephrotic syndrome, or Kawasaki disease, if they had a history of previous coronary interventions, or if their lipid profiles were incomplete. As a result, 4644 consecutive postmenopausal women were included in this study, of which 3039 CAD were patients, and 1605 were non-CAD subjects. The flow chart of the selection process is shown in Fig. 1.

The Declaration of Helsinki was observed when designing this study, which was approved by the Ethics Committee of the Beijing Anzhen Hospital. As the data were obtained retrospectively from electronic medical records, no written informed consent was obtained from the participants.

### Definition of CAD

All patients underwent CAG, and the results were assessed by two experienced cardiologists unaware of this study. CAD was defined according to the WHO diagnostic standard established in 1979. The number of diseased coronary arteries was calculated as the total count of main coronary arteries stenosed ≥50%, including left main trunk, left anterior descending branch, left circumflex branch, right coronary artery, and main branch (vessel diameter > 2.0 mm). The control group lacked typical angina symptoms, while the main coronary artery stenosis was less than 50%.

### Laboratory assessments

Upon patient inpatient admission, samples of peripheral venous blood were collected for testing. In addition, patient demographic and clinical information was collected including basic vitals and age, height, sex, weight, history of alcohol use, history of smoking, history of EH and DM, and family history of heart disease, with this information being obtained from EMR for the present study. Recurrent systolic blood pressure ≥ 140 mmHg and / or diastolic blood pressure ≥ 90 mmHg that had been measured twice of more was defined as EH, while DM was defined as fasting blood glucose ≥7.0 mmol/L, random glucose level ≥ 11.1 mmol/L, or a previous DM diagnosis. Laboratory data including fasting blood glucose, TC, LDL-C, HDL-C, and TG, were obtained by automatic biochemical analyzer. AIP was determined as being the log_10_(TG/HDL-C).

### Statistical analysis

SPSS 22.0 (SPSS Inc., IL, USA) was used for statistical testing. Continuous data are means ± standard deviation (SD). When normally distributed, these data were compared using Student’s t-tests or one-way ANOVAs. Nonparametric tests were used to analyze data not conforming to the normal distribution. Ordinal variables are given as frequencies or percentages and were compared via chi-squared tests. Pearson correlation analyses were used to compare the relationship between AIP and other variables. Multivariate logistic regression analysis was conducted to evaluate the association between AIP and the risk for CAD, and was expressed as odds ratios (ORs) with 95% confidence intervals (95% CIs). *P* < 0.05 was the significance threshold.

## Results

### Basic characteristics

Study patient baseline characteristics are compiled in Table 1. The investigation included 4644 subjects; 3039 (65.44%) were CAD patients and 1605 (34.56%) were controls. The average age of the patients was 64.18 ± 7.57 years. CAD group patients were significantly older than controls, and their BMI was higher. The CAD group also had fractions of smokers and patients with the history of EH and DM that were significantly higher.

**Table 1:** Baseline characteristics of involved participants

	Total (*n* = 4644)	nonCAD (*n* = 1605)	CAD (*n* = 3039)	*P* value
Age, years	64.18 ± 7.57	62.97 ± 7.43	64.82 ± 7.57	<0.001
BMI, kg/m^2^	24.71 ± 3.01	23.77 ± 2.15	25.21 ± 3.33	<0.001
SBP, mmHg	128.00 ± 13.65	129.17 ± 15.40	127.38 ± 12.58	<0.001
DBP,mmHg	72.97 ± 9.12	73.71 ± 9.64	72.58 ± 8.81	<0.001
Smoke, n(%)	330(7.11)	91(5.67)	239(7.86)	0.006
Drinking, n(%)	44(0.95)	17(1.06)	27(0.89)	0.568
Medical history, n(%)				
DM, n (%)	1423(30.64)	380(23.68)	1043(34.32)	<0.001
EH, n (%)	3015(64.92)	923(57.51)	2092(68.84)	<0.001
Hyperlipidaemia, n(%)	1777(38.26)	557(34.70)	1220(40.14)	<0.001
Family history of CVD, n(%)	252(5.43)	83(5.17)	169(5.56)	0.577
Laboratory results				
TC, mmol/L	4.47 ± 1.08	4.48 ± 1.04	4.45 ± 1.11	0.352
TG, mmol/L	1.73 ± 1.17	1.66 ± 1.12	1.77 ± 1.20	0.003
LDL, mmol/L	2.63 ± 0.84	2.63 ± 0.86	2.62 ± 0.89	0.849
HDL, mmol/L	1.12 ± 0.26	1.15 ± 0.27	1.09 ± 0.25	<0.001
Non-HDL	3.33 ± 1.04	3.30 ± 0.99	3.35 ± 1.07	0.141
TC/HDL	4.17 ± 1.19	4.11 ± 1.17	4.20 ± 1.99	0.017
LDL/HDL	2.44 ± 0.90	2.42 ± 0.90	2.45 ± 0.91	0.283
LCI	22.11 ± 24.63	21.48 ± 23.52	22.45 ± 25.19	0.201
AI	3.17 ± 1.19	3.11 ± 1.17	3.20 ± 1.20	0.017
AIP	0.14 ± 0.28	0.10 ± 0.28	0.15 ± 0.28	<0.001
Medical treatment, n(%)				
Aspirin	4240(91.30)	1326(82.62)	2914(95.89)	<0.001
Clopidogrel	2736(58.91)	393(24.49)	2343(77.10)	<0.001
Statin	3693(79.52)	933(58.13)	2760(90.82)	<0.001
β-blocker	1446(31.14)	747(46.54)	699(23.00)	<0.001
ARB	505(10.87)	270(16.82)	235(7.73)	<0.001
ACEI	162(3.49)	91(5.67)	71(2.34)	<0.001

Values are given as mean ± standard deviation, medians with interquartile range or number (%)

*CAD* Coronary artery disease, *BMI* Body mass index, *SBP* Systolic blood pressure, *DBP* Diastolic blood pressure, *DM* Diabetes mellitus, *EH* Essential hypertension, *CVD* Cardiovascular Disease, *TC* Total cholesterol, *TG* Triglyceride, *HDL* High-density lipoprotein cholesterol, *LDL* Low-density lipoprotein cholesterol, *LCI* Lipoprotein Combine Index, *AI* Atherogenic Index, *AIP* Atherogenic index of plasma, *ARB* Angiotensin receptor blockers*, ACEI* Angiotensin-converting enzyme inhibitor

Bold values indicate statistical significance

CAD patients had significantly higher serum TC level than in control subjects. Conversely, their HDL-C levels were lower. CAD patients had higher non-traditional lipid profile values, including TC/HDL-C, AI, and AIP, relative to controls.

### The relationship between AIP and other variables

The relationship between AIP and other continuous variables was assessed via a Pearson correlation analysis. As shown in Table 2, AIP was positively correlated with TC (r = 0.157), TG (r = 0.835), LDL-C (r = 0.058), non-HDL-C (r = 0.337), TC/HDL-C (r = 0.683), LDL-C/HDL-C (r = 0.437), LCI (r = 0.662), and AI (r = 0.684) (all *P* < 0.001). AIP was negatively correlated with HDL-C (r = − 0.682, *P* < 0.001), but was independent of age (r = − 0.022; *P* = 0.130) and BMI (r = 0.020, *P* = 0.168).

**Table 2:** Correlation between AIP and other variables

Variable	R	*P*
Age	−0.022	0.130
BMI	0.020	0.168
TC	0.157	<0.001
TG	0.835	<0.001
LDL	0.058	<0.001
HDL	−0.682	<0.001
Non-HDL	0.337	<0.001
TC/HDL	0.683	<0.001
LDL/HDL	0.437	<0.001
LCI	0.662	<0.001
AI	0.684	<0.001

*BMI* Body mass index, *TC* Total cholesterol, *TG* Triglyceride, *LDL* Low-density lipoprotein cholesterol, *HDL* High-density lipoprotein cholesterol, *LCI* Lipoprotein Combine Index, *AI* Atherogenic Index

### Logistic regression analysis

**Fig. 2:**
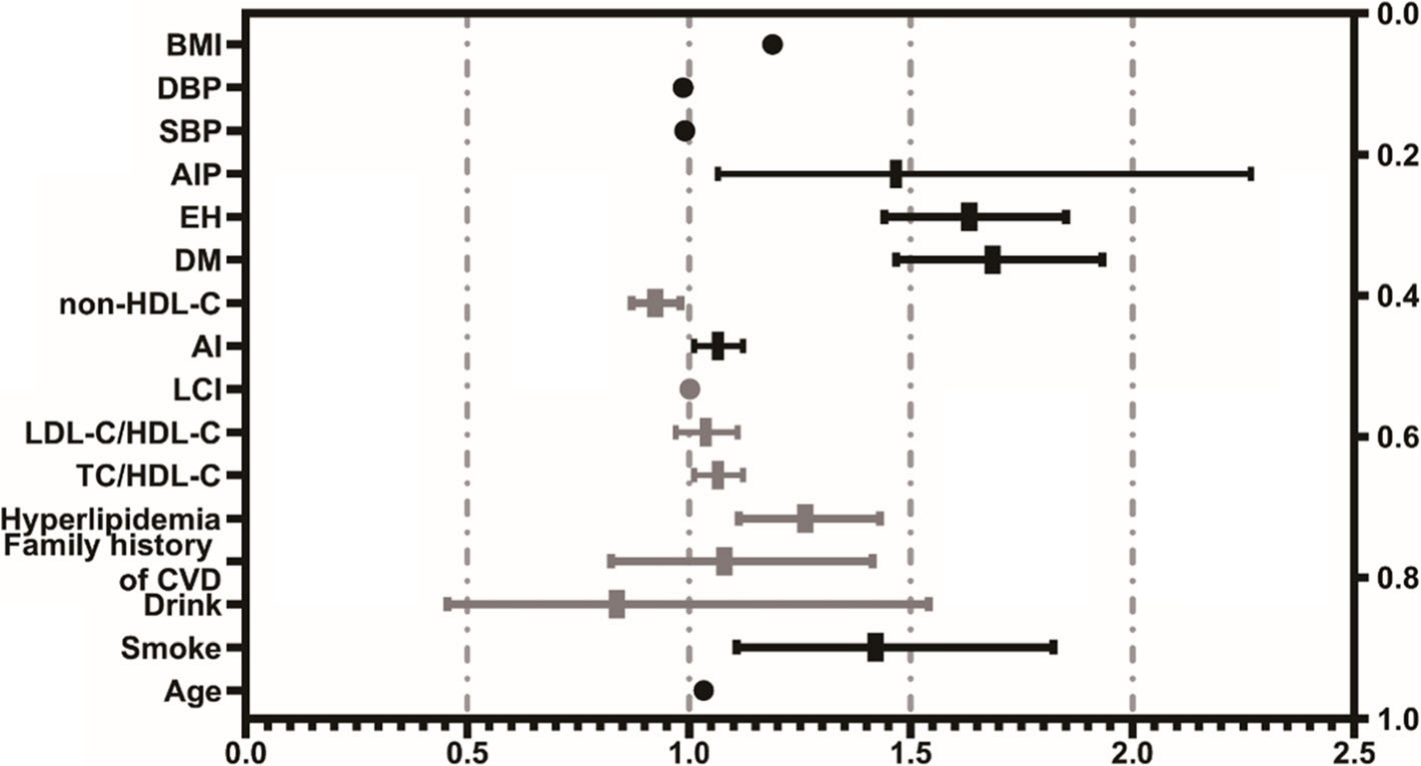
Forest plot of Univariate analysis of CAD risk factors. *CAD* coronary artery disease, *BMI* body mass index, *DBP* diastolic blood pressure, *SBP* systolic blood pressure, *AIP* atherogenic index of plasma, *EH* essential hypertension, *DM* diabetes mellitus, *HDL-C* high-density lipoprotein cholesterol, *TC* total cholesterol, *CVD* Cardiovascular Disease

**Fig. 3:**
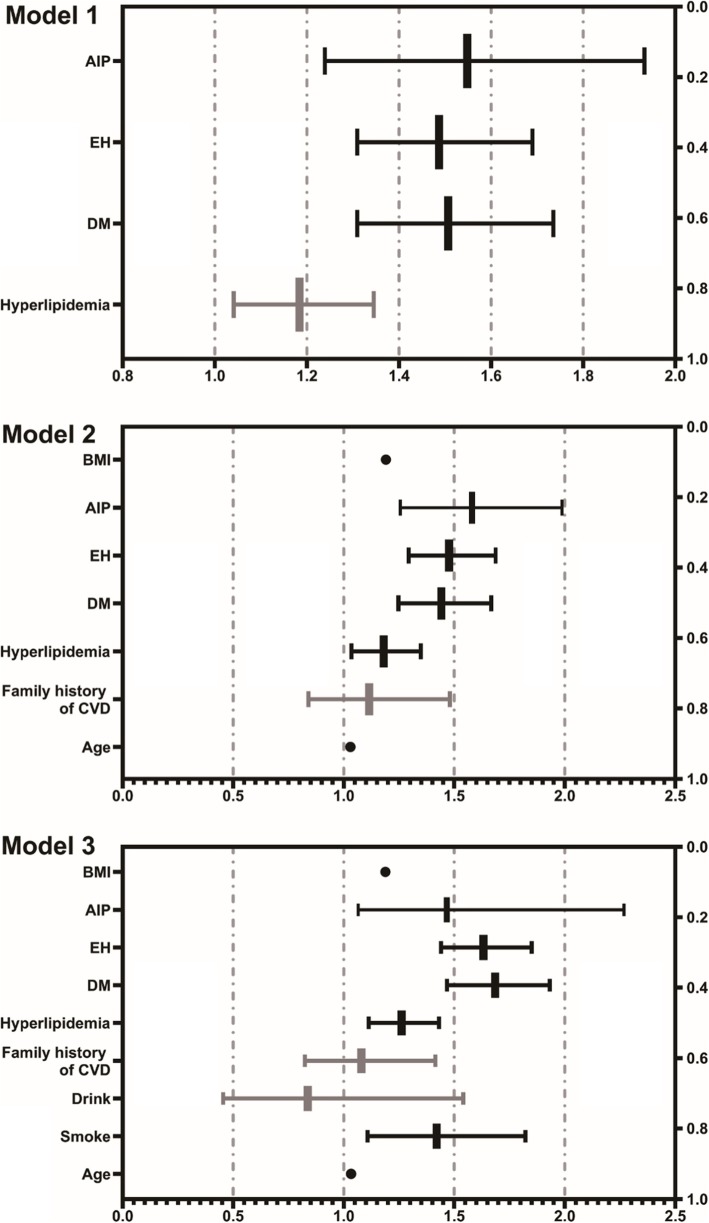
Forest plot of Multivariate logistic regression analysis model of different CAD risk factors. Model 1, adjusted for EH, DM and hyperlipidemia; Model 2, adjusted for confounders in model 1 plus BMI, family history of CVD and age; Model 3, adjusted for confounders in model 2 plus drinking and smoking. *CAD* coronary artery disease, *AIP* atherogenic index of plasma, *EH* essential hypertension, *DM* diabetes mellitus, *BMI* body mass index, *CVD* Cardiovascular Disease

A univariate logistic regression analysis revealed AIP to be a lipid parameter closely related to CAD with unadjusted OR 1.824 (95% CI: 1.467–2.267, *P* < 0.001) (Fig. 2). Multivariate logistic regression analysis was carried out to eliminate the interference of confounding factors. In Model 1, after adjusting for typical clinical prognostic factors including DM, hypertension, and hyperlipidemia, AIP was independently associated with CAD risk (OR 1.548; 95% CI: 1.239–1.933, *P* < 0.001). In Model 2, after adjusting for known CAD risk factors (age, BMI, EH, DM, hyperlipidemia, and family history of CVD), the association between AIP and CAD continued to be present (OR 1.580; 95% CI: 1.256–1.988, *P* < 0.001). In Model 3, after adjusting for the same CAD risk factors as in Model 2 plus drinking and smoking, AIP was still a strong risk factor for CAD (OR 1.553; 95% CI: 1.234–1.955, *P* < 0.001) (Fig. 3).

## Discussion

The evaluation of AIP in postmenopausal women has been rarely performed and the present investigation is the first large-scale study we are aware of to assess the relationship between AIP and CAD risk in postmenopausal (50 years and older) Chinese Han women. The performed analyses demonstrated that AIP is an independent risk factor for CAD in postmenopausal women in China, and its predictive value may be superior to that of traditional lipid profile.

Epidemiological studies show that the incidence of CVD in age-matched premenopausal women is about twice lower than that in men, but the incidence of CVD in postmenopausal women is comparable in both genders [15]. Estrogen replacement therapy reduces the incidence of atherosclerosis in postmenopausal women to the premenopausal level, demonstrating that estrogen protects women against CVD [16]. The anti-atherosclerotic effect of estrogen is mostly attributed to its impact on blood lipid concentration [17]. Therefore, the blood lipid level may play a more important role in the onset and development of CAD in postmenopausal women. The current study involved 4644 participants, including 1605 non-CAD subjects and 3039 CAD patients. Among postmenopausal women, the prevalence of diabetes and hypertension in smokers was significantly higher than in non-smokers.

A large body of evidence indicates that dyslipidemia, defined by conventional lipid profile including TC, LDL-C, TG and HDL-C, is the main risk factor for CAD [18]. Compared with LDL, small density LDL (sdLDL) invades the arterial wall and forms deposits more easily. sdLDL is also more prone to be oxidized, forming an oxidized low-density lipoprotein (oxLDL). When oxLDL is phagocyted by macrophages, they are transformed into foam cells that produce atherosclerosis and CVD. In 2002, the National Cholesterol Education Program identified sdLDL as a major risk factor for CAD and recommended the detection of sdLDL as a part of blood lipid testing [19]. However, due to the complexity and high cost of this method, its wide application in the clinical setting is difficult to promote. At the same time, the level of sdLDL is accurately reflected by AIP, which can be calculated directly as the logarithm of TG to HDL-C molar ratio [5]. Therefore, AIP can be considered as a cost-effective and reliable index for predicting CAD.

AIP has been found to be linked with metabolic syndrome and increases in BMI in several studies [20, 21]. In 2015, Zhu and collaborators demonstrated AIP is positively correlated with DM [22]. More recently, AIP was identified as a powerful predictor of the risk of fatty liver [23]. Additionally, AIP correlates positively with serum uric acid, C-reactive protein, and oxidative stress [24, 25]. An increasing amount of evidence shows that AIP is a strong indicator of the risk for CAD [9, 13, 14]. Likewise, the present study documented a positive correlation between AIP and CVD risk. In addition, after adjusting for age, BMI, smoking, drinking, EH, DM, hyperlipidemia, and family history of CVD, there was still a positive and significant correlation between AIP and CVD risk.

The average AI*P* value (0.14 ± 0.28) obtained in the current work is similar to that found by Nwagha [12], and lower than that in postmenopausal women in Xinjiang, China (average 0.20 ± 0.27) [13]. This discrepancy is consistent with previous reports documenting that the AIP values vary greatly among different ethnic groups [26]. In a previous study on the middle-aged population in China, the AIP value was 0.092 ± 0.325 [21], while in the Slovak population aged 40, the AIP value was 0.064 ± 0.310 in males and 0.150 ± 0.306 in females [26]. These data, in combination with the results of the present work, indicate that the AIP value may be greater in women. Similarly, we have found a positive correlation between AIP and known lipid parameters associated with smoking, diabetes, hypertension, and other risk factors for CVD. Therefore, the possibility can be raised that interventions reducing these parameters may lead to a decrease in AIP values [27]. Therefore, postmenopausal women should be encouraged to adopt a healthy lifestyle.

The current investigation documented that AIP is not related to age, a finding consistent with the conclusion of Nansseu and colleagues [12]. One previous analysis of a normal Chinese population showed AIP to increase with age [9]. This discrepancy may be due, at least in part, to the differences in the population included in the studies. Here, the average age of subjects was 64.18 ± 7.57 years, with all of these subjects having been subjected to hospitalization and having undergone CAG. More attention is often paid to factors such as the control of cholesterol intake and the maintenance of a healthy lifestyle in the elderly, which may account for the observed difference.

Wu and coworkers have found that the AIP of postmenopausal women with CAD in Xinjiang was higher than in the control group [13]. However, Nansseu and collaborators have shown that AIP is not an independent predictor of CVD in postmenopausal women in Cameroon [12]. In another study of the Han Chinese population, AIP was found to be independently associated with ACS risk in men only [28]. Therefore, whether AIP can be used as a biomarker for predicting CAD in postmenopausal women remains controversial. It should be indicated, however, the present investigation has several advantages in comparison with the above-discussed studies. First, the sample size was significantly larger (4644 vs. 754 vs. 108). Second, this study both assessed the relevance of AIP values to CAD risk and compared the relative utility of AIP and other CAD-related lipid parameters. The current study documented that the AIP value in the CAD group was higher than in the control group (0.15 ± 0.280 vs 0.10 ± 0.28). Univariate logistic regression revealed AIP to be the lipid parameter most strongly correlated with CAD, and the unadjusted OR was 1.824 (95% CI: 1.467–2.267, *P* < 0.001). In addition, multiple logistic regression model indicated that AIP is the most powerful predictor of CAD. Finally, the correlation analysis demonstrated that AIP was most positively correlated with TG (r = 0.835, *P* < 0.001), and negatively correlated with HDL-C (r = − 0.683, *P* < 0.001).

In conclusion, our work documented that AIP may be a powerful and independent predictor of CAD in Han Chinese postmenopausal women.

### Limitation

Some limitations of this study must be acknowledged. Due to its retrospective design, some relevant data, such as waistline, physical exercise, lifestyle, and eating habits could not be obtained. Given that these data are associated with AIP; this limitation might have affected the obtained results. In addition, since the participants visited the hospital to obtain angiography, they might have had high CVD risk. Therefore, it is necessary to carry out a broader, community-based, and well-designed prospective study to better understand the relationship between AIP and CVD risk, particularly in postmenopausal women.

## Conclusion

Our study demonstrated that AIP may be a powerful and independent predictor of CAD in the population of Chinese Han postmenopausal women, and may be superior to traditional lipid indices.

## Data Availability

The datasets used and/or analyzed during the current study will be available from the corresponding author on reasonable requests.

## Abbreviations

ACS: Acute coronary syndrome
AI: Atherogenic index
AIP: Atherogenic index of plasma
ALT: Glutamic-pyruvic transaminase
ANOVA: Analysis of variance
AST: Glutamic-oxalacetic transaminase
AUC: Area under the curve
BG: Blood glucose
BMI: Body mass index
CAD: Coronary artery disease
CAG: Coronary angiography
CI: Confidence interval
CVD: Cardiovascular disease
DBP: Diastolic blood pressure
DM: Diabetes mellitus
EH: Essential hypertension
EMR: Electronic medical record
FL: Fatty liver
HDL: High density lipoprotein
HDL-C: High-density lipoprotein cholesterol
LCI: Lipoprotein combine index
LDL-C: Low-density lipoprotein cholesterol
OR: Odds ratio
ROC: Receiver operating characteristic
SBP: Systolic blood pressure
SD: Standard deviation
TC: Total cholesterol
TG: Triglyceride
VLDL-C: Very low-density lipoprotein cholesterol
WHO: World Health Organization
